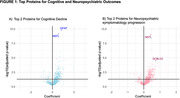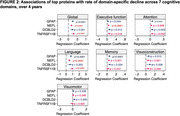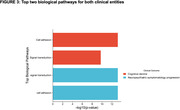# Blood‐based Proteomic Profiling for the Progression of Cognitive Decline, Neuropsychiatric Symptomatology, and Neuroimaging Outcomes within an Asian Memory Clinic Cohort

**DOI:** 10.1002/alz.094884

**Published:** 2025-01-09

**Authors:** Ming Ann Sim, Hyung Won Choi, Yuan Cai, Arthur Mark Richards, Christopher Chen

**Affiliations:** ^1^ National University Hospital, Singapore, Singapore Singapore; ^2^ National University of Singapore, Singapore, singapore Singapore; ^3^ National University of Singapore, Singapore, Singapore Singapore; ^4^ Division of Neurology, Department of Medicine and Therapeutics, Faculty of Medicine, The Chinese University of Hong Kong, Prince of Wales Hospital, Hong Kong SAR Hong Kong; ^5^ The Chinese University of Hong Kong, Hong Kong Hong Kong; ^6^ Department of Medicine, Yong Loo Lin School of Medicine, National University of Singapore, Singapore Singapore; ^7^ Christchurch Heart Institute, University of Otago, New Zealand, Christchurch New Zealand; ^8^ Cardiovascular Research Institute, National University Health System, Singapore, Singapore Singapore; ^9^ National University of Singapore, Kent Ridge Singapore; ^10^ Memory Aging and Cognition Center, National University Health System, Kent Ridge Singapore; ^11^ Memory Aging and Cognition Center, National University Health System, Singapore Singapore; ^12^ Yong Loo Lin School of Medicine, National University of Singapore, Singapore Singapore

## Abstract

**Background:**

The prognostic performance of plasma proteomics for longitudinal changes in (i)cognition (ii)neuropsychiatric symptomatology and (iii)neuroimaging outcomes within South East Asia, where prevalence of cerebrovascular disease is high, remains un‐investigated.

**Methods:**

A prospective cohort of Singaporean memory clinic attendees was followed‐up for 4 years. Annual neurocognitive and neuropsychiatric assessments were performed alongside 2‐yearly brain magnetic resonance imaging (MRI) scans. Incident cognitive decline was defined as a Clinical Dementia Rating Scale Sum‐of‐Boxes (CDR‐SB) ≥3 increment from baseline. Significant neuropsychiatric symptomatology progression was defined by a Neuropsychiatric Inventory score increment of ≥4 from baseline.

Neuroimaging outcomes evaluated included (i)rates of progression of cerebral small vessel disease (white matter hyperintensity volume) and (ii)hippocampal atrophy on 2‐yearly MRI scans.

1536 plasma proteins (encapsulating all major biological pathways including neurological, immune‐oncological, cardiovascular and inflammatory pathways) were profiled at baseline using the Olink Explore 1536 platform. Their associations with cognitive, neuropsychiatric and MRI outcomes were subsequently evaluated in multivariable regression models, with correction for false‐discovery rate.

**Results:**

Of 528 subjects (mean age 72.83±7.81years, 43.8% male, 57.9% cerebrovascular disease, 22.4% NCI, 40.3% CIND, 37.3% dementia), the incidence of cognitive decline was 31.6% (167/528). Among 245 proteins significantly associated with cognitive decline, GFAP and NEFL were the most powerfully prognostic (Fig. 1).

The incidence of significant progression of neuropsychiatric symptomatology was 55.68% (294/528). Among 310 proteins significantly associated with the progression of neuropsychiatric symptoms, NEFL and DCBLD2 were the most prognostic (Fig. 1).

NEFL and TNFRSF11B were the most significantly dysregulated proteins for cerebral small vessel disease progression, while GFAP and NEFL significantly associated with rate of hippocampal atrophy. These top proteins also significantly associated with the rates of domain‐specific decline in 4 or more cognitive domains assessed over 4 years (Fig. 2).

The top two gene ontology biological pathways identified from all data on significantly dysregulated proteins, were (i)cell adhesion and (ii)signal transduction, in both clinical entities (Fig. 3).

**Conclusion:**

Our findings, upon interrogation of a biobank from a well‐annotated cohort, identifies late‐stage protein biomarkers and biological pathways for a suite of longitudinal cognitive, neuropsychiatric and neuroimaging outcomes. These biomarkers warrant further validation and mechanistic elucidation as potential therapeutic targets.